# The frequency of smoking and problem drinking among general hospital inpatients in Brazil -using the AUDIT and Fagerström questionnaires

**DOI:** 10.1590/S1516-31802000000500005

**Published:** 2000-09-01

**Authors:** Neliana Buzi Figlie, Sandra Cristina Pillon, John Dunn, Ronaldo Laranjeira

**Keywords:** AUDIT, Fagerström, Screening, Liaison psychiatry, In-patients, AUDIT, Fagerström, Instrumento de rastreamento, Interconsulta, Pacientes internados

## Abstract

**CONTEXT:**

Although the CAGE questionnaire is one of the most widely used alcohol screening instruments, it has been criticized for not identifying people who are drinking heavily or who have alcohol related problems but do not as yet show symptoms of alcohol dependence. The AUDIT (Alcohol Use Disorder Identification Test) questionnaire was developed by WHO as a screening instrument specifically designed to identify problem drinkers, as well as those who were already dependent on alcohol.

**OBJECTIVE:**

The aim of this study was to use the AUDIT and Fagerström questionnaires in a general hospital inpatient population to measure the frequency of problem drinking and nicotine dependence, and to see if levels varied between medical speciality.

**DESIGN:**

Retrospective cross-sectional study.

**SETTING:**

Federally funded public teaching hospital.

**SAMPLE:**

275 inpatients from both genders.

**MAIN MEASUREMENTS:**

Socio-demographic data, AUDIT (Alcohol Use Disorders Identification Test) and Fagerström Test for Nicotine Dependence.

**RESULTS:**

We interviewed 275 inpatients, 49% of whom were men and 51% women. Thirty-four patients were identified as “cases” by the Audit questionnaire; 22% of the male patients and 3% of the females. Just over 21% of inpatients were current smokers. The gastroenterology (26%) and general medicine (16%) inpatient units had the largest number of individual cases.

**CONCLUSIONS:**

Only by knowing the prevalence of alcohol abuse/dependence and nicotine dependence in a general hospital can we evaluate the need for a specialized liaison service to identify and treat these patients.

## RESUMO

**CONTEXTO:**

Embora o CAGE seja um dos instrumentos de rastreamento do consumo alcoólico mais utilizado, o mesmo tem sofrido críticas por não identificar pessoas que possuem um consumo nocivo ou pessoas que apresentam problemas relacionados ao consumo alcoólico sem ter desenvolvido um quadro de dependência alcoólica. O questionário AUDIT (Alcohol Use Identification Test) foi desenvolvido pela Organização Mundial de Saúde como instrumento de rastreamento especificamente para identificar pessoas com consumo nocivo do álcool, como também aquelas que possuem dependência do álcool.

**OBJETIVO:**

Mensurar a freqüência de uso nocivo/dependência de álcool e nicotina através do AUDIT e Fagerström.

**TIPO DE ESTUDO:**

Estudo de corte transversal retrospectivo.

**LOCAL:**

Hospital escola do serviço público federal, São Paulo, Brasil.

**AMOSTRA:**

275 pacientes internados de ambos sexos.

**VARIÁVEIS ESTUDADAS:**

Dados sócio-demográficos, AUDIT e Fagerström Test for Nicotine Dependence.

**RESULTADOS:**

Foram entrevistados 275 pacientes que se encontravam internados, sendo que 49% destes eram homens e 51% mulheres. 34 pacientes foram identificados como “casos” através do AUDIT, que corresponderam a 22% dos pacientes do sexo masculino e 3% do sexo feminino. Apenas 21% dos pacientes foram considerados fumantes. As enfermarias de gastroenterologia (26%) e clínica médica (16%) foram consideradas como detentoras de maior número de casos quando comparadas com as outras enfermarias.

**CONCLUSÕES:**

Somente com o conhecimento da prevalência de uso nocivo/dependência de álcool e de nicotina, em um hospital geral, é possível estimar a necessidade de um serviço de interconsulta que pudesse identificar e tratar esses pacientes.

## INTRODUCTION

Data from the USA indicate that only 15% of alcoholics seek specialized treatment for alcoholism. However, 70% of the approximately 11 million alcoholics in that country have been treated in general medicine services within the last 6 months.^1^ Most of these visits are for general medical assessments and not for mental health problems. Studies calculate that the economic costs of alcohol and drug abuse and mental illness are estimated at US$ 273.3 billion. The estimates include US$ 85.8 billion for alcohol abuse, US$ 58.3 billion for drug abuse and US$ 129.3 billion for mental illness.^2^

Over the last two decades several studies have been undertaken in Brazil that show a relatively high prevalence of alcohol misuse among inpatients in general hospitals. One of the pioneer studies in this field was that of Masur et al,^3^ who found that 55% of male inpatients on a general medical ward consumed more than half a liter of “cachaça” (a distilled spirit made from sugar cane) per day. Since then other authors, from different regions of the country, have found a prevalence of alcohol abuse or dependence of between 9% and 32% among general hospital inpatients.^4–7^ Much of this variation in reported prevalence is due to methodological differences between the studies, in particular the definition of alcohol dependence or abuse. Levels of consumption are, by and large, considerably higher than those found in the general population. For example, in the Brazilian Multicentric Psychiatric Morbidity Survey, conducted in three major urban areas in Brazil and using DSM-III diagnostic criteria, the prevalence of alcohol dependence was 15%.^8^

Research from other countries largely supports the finding that inpatients are more likely to have problems with alcohol.^9–11^ The importance of identifying these patients lies in the possibility of providing some form of intervention aimed at reducing alcohol consumption and thereby reducing the risk that the patient will subsequently develop complications of alcohol misuse.

Just how and by whom these patients should be identified is open to debate. Short screening instruments have the advantage that they do not take long to fill in and can be used with very little training by almost any health care professional.^12^ The CAGE questionnaire is one of the most widely used alcohol screening instruments and has a high sensitivity and specificity for identifying patients with alcohol dependence. However, it has been criticized for not identifying people who are drinking heavily or who have alcohol-related problems but do not as yet show symptoms of alcohol dependence. Indeed it is these very patients who may benefit most from early therapeutic interventions. The AUDIT (Alcohol Use Disorder Identification Test) questionnaire was developed by WHO^13^ as a screening instrument specifically designed to identify problem drinkers, as well as those who were already dependent on alcohol. It has a reported sensitivity of 92% and a specificity of 93%.^14^ Skipsey, et al.^15^ found an excellent level of internal consistency in the identification of “hard” drinkers and alcohol dependence. When compared with the MAST (Michigan Alcoholism Screening Test), the AUDIT was considered superior, as the MAST failed to identify recent problems brought on by heavy alcohol consumption. The AUDIT has been found to be a very promising instrument for the identification of alcohol abuse and dependence among patients in diverse medical settings.^16–18^

It is estimated that 30% of men and 35% woman are current smokers and about 3 million people die each year due to consequences of cigarette smoking. According to the World Health Organization, if this prevalence is maintained, by the year 2020 ten million people will be dying per year from smoking-related diseases.^19^

Nicotine dependence represents a serious public health problem. Data show that 19% of deaths in the USA are smoking–related.^20^ In Brazil there is a lack of data concerning the prevalence of smoking, especially among populations in contact with health care services.

The Fagerström Tolerance Questionnaire (FTQ) is a short self-reported measure of nicotine dependence.^21^ The FTQ has been criticized because of psychometric deficiencies, such as having a multifactorial structure, a low level of reliability and poor item selection.^22^ Heatherton et al^23^ revised the questionnaire to produce what they call the Fagerström Test for Nicotine Dependence (FTND), which has an internal consistency of 0.61 and whose scores are closely related to biochemical indices of heavy smoking. Saxon, et al.^24^ found that when smokers were assessed with both instruments, the mean scores on the FTND were higher than those of the FTQ.

The aim of this study was to use the AUDIT and Fagerström questionnaires in a general hospital inpatient population to measure the frequency of problem drinking and nicotine dependence, and to see if levels varied between medical speciality.

## METHODS

### Design

This study is a retrospective cross sectional study, which the data was collected over two days in via a structured interview with all inpatients at that time.

### Setting

The study was undertaken at the Hospital São Paulo / Escola Paulista de Medicina, a federally funded public teaching hospital.

### Participants

Patients were interviewed over two consecutive days in July 1995. The hospital has 512 beds, of which 394 were included in the study, with the remainder being excluded because they formed part of inpatient units that treated only children and teenagers below the age of 16. The in-patient units included were: psychiatry, general medicine, intensive care unit, orthopedics, gastroenterology, cardiology, urology, ophthalmology, neurology, obstetrics and gynecology, infectious diseases and parasitology unit, renal unit, hematology, endocrinology, surgical units and pulmonary diseases unit. Thirty patients were excluded because they were under the age of 16, a further 7 refused to participate and 82 were physically or psychologically unable to consent, primarily because they were unconscious, confused or drowsy. This gave a final sample of 275 patients. Interviews were performed at the patient's bedside and involved a team of 9 interviewers who had been trained in the use of the questionnaire.

### Instruments

The interview took at most 10 minutes to complete and consisted of closed response questions, either multiple choice or dichotomous. The first part of the instrument covered socio-demographic data, including age, sex, level of schooling, occupation and family income. The second part was a Portuguese version of the AUDIT, which had been translated by R. L. (an English-speaking Brazilian psychiatrist) and checked then by J. D. (a Portuguese-speaking English psychiatrist). All items of the AUDIT refer to the last 12 months. The final score ranges 0 to 40, with a score over 8 indicating that the person probably has an alcohol problem. In addition, further questions were asked about alcohol problems preceding the last 12 months and previous treatment experiences. The third part of the questionnaire consisted of a Portuguese version of the Fagerström Test for Nicotine Dependence - FTND. This instrument consist of 6 questions concerning the respondent's current smoking behavior, with scores ranging from 0 to 10, and with scores classified into mild, moderate and severe dependence. The final part of the instrument concerned current and past cigarette smoking.

### Statistical Methods

Data were entered into the SPSS program and analyzed using descriptive statistics.

## RESULTS

Forty-nine percent of the sample were male and the average age was 42 (SD 17, range: 16 to 85). The demographic characteristics of the patients are shown in Table 1.

**TABLE 1:** Socio-demographic data of inpatients from a general hospital in São Paulo, Brazil (n = 275)

Characteristic	Male n=135 (%)	Female n=140 (%)
**Age**		
mean in years	42	39
**Marital Status**		
Single	40 (30)	34 (24)
Married/ Living Together	83 (62)	80 (57)
Divorced/Separated	7 (5)	12 (9)
Widowed	5 (4)	14 (10)
**Level of schooling**		
Illiterate	11 (8)	9 (6)
“1st grade” (8 years of schooling)	91 (67)	94 (67)
“2nd grade” (high school)	17 (13)	30 (21)
College/University	16 (12)	7 (5)
**Occupation**		
Unemployed	5 (4)	8 (6)
Blue collar job	35 (26)	18 (13)
White collar job	27 (20)	23 (16)
Pensioned/Student	65 (48)	35 (25)
Other	3 (2)	56(40)
**Family income**		
1 to 5 m.w.*	81 (60)	88 (63)
5 to 20 m.w.	35 (26)	25 (18)
Up to 20 m.w.	4 (3)	0
Don't know	15 (11)	27 (19)

* minimum wage = R$ 114.00.

Of the 275 patients interviewed, 34 (12.4%; 95% CI: 8.5 to 16.3%) were diagnosed as having an alcohol use disorder by the AUDIT questionnaire. Among men the prevalence was 22% (n = 30) and woman 3% (n = 4). The gastroenterology and general medical inpatient units had the largest number of individual cases: gastroenterology 11 (26%) and general medicine 5 (16%). Several wards had no cases at all: orthopedics, hematology, endocrinology, psychiatry and neurology.

Of the total sample, 70% of patients admitted that they had drunk more heavily in the past than during the last year, with 7% saying that they had had a period in their life in which they had drunk on a daily or almost daily basis. Just over 46% of these latter patients scored positive on the AUDIT. Only two percent of AUDIT positive patients said that they had undergone some form of alcohol treatment in the past, with Alcoholics Anonymous (AA) and inpatient treatment being most commonly cited.

Twenty-one percent of patients were current smokers but a further 32% were ex-smokers of varying duration, whilst 47% said that they had never smoked.

Regarding the frequency of smoking by ward, gastroenterology had the highest level of smokers (n = 11, 23%) followed by general medicine (n = 8, 14%). Using the Fagerström Test for Nicotine Dependence, 30% of smokers were classified as having mild nicotine dependence, 44% moderate and 26% severe. About 57% reported a period of higher cigarette consumption compared to their current level. Forty-eight percent had already tried to stop smoking without success and 27% had never tried.

## DISCUSSION

Only by knowing the prevalence of alcohol abuse/dependence and nicotine dependence in a general hospital can we evaluate the need for a specialized liaison service to identify and treat these patients. Our study shows that the frequency of AUDIT positive case was 12% and that 21% of patients were current smokers. Alcohol abuse was more frequent among men and among patients on the gastroenterology and general medical wards.

The AUDIT questionnaire is simple and quick to use. Its application does not require any specialized training, a fact that facilitates its use in a variety of settings including developing countries where highly trained staff are in short supply.^25–27^ If the AUDIT were used as a screening instrument on admission to hospital, potential cases of alcohol abuse could be referred to a specialized liaison service. As mentioned above, the advantage of the AUDIT is that it can identify patients before alcohol dependence has developed, so that an intervention can be offered before the patient has developed severe complications of alcohol dependence, i.e. as a form of secondary prevention. Also, in a country like Brazil, where cash constraints are tight, such an intervention is relatively cheap and may save money in the long term.

The FTND has been shown to be a reliable measure of nicotine dependence and is also a predictor of success in smoking cessation. It can also be used to titrate nicotine dosage against severity of dependence in nicotine replacement programs designed to help severely dependent smokers to quit.^28^ It has been estimated that if health workers spend three to five minutes advising patients about the advantages of stopping smoking and give them self-help materials to reinforce this advice, 5 to 8% of smokers will quit smoking within 12 months.^29,30^ Although this number is quite low, the high turnover on general medical wards means that a large number of patients could be treated over a space of one year.

The National Cancer Institute has developed an office-based intervention for physicians and other health care professionals that provides a brief but effective intervention based on the four “A's”: 1. Ask all patients about their smoking behavior; 2. Advise every smoker to stop smoking; 3. Assist each smoker in setting a stop date; and 4. Arrange a follow-up visit.^31^ This intervention could be adapted to an inpatient setting and could also be applied to alcohol abuse.

We found the highest frequency for both alcohol abuse and nicotine dependence was on the gastroenterology and general medicine wards, although the absolute numbers involved were small. However, this could be an indication that a liaison service directed towards identifying and treating alcohol abuse and smoking would be more cost effective, and reach a greater number of cases, if implemented on these wards.

There are several limitations to this study. First, only patients from one hospital in São Paulo were screened, and therefore we cannot generalize our findings to other types of hospital or other regions of the country where demographic characteristics and health service provision may differ. Second, this study was performed on two days in the winter month of July. Although the subtropical winter in São Paulo is not severe, there may still be seasonal variations in diseases that lead to hospital admission, such as chest infections. This could have an effect on the proportion of patients admitted to hospital who were smokers or alcohol abusers. However, the only seasonal variation in hospital admissions reported in São Paulo are for children with rotavirus infection and respiratory disease,^32–34^ who would not have been eligible for this study. The fact that there were no cases of alcohol abuse on the psychiatric ward is probably related to the fact that only women are admitted to this unit, among whom the prevalence of alcohol abuse would be low. No attempt was made to relate the use of alcohol or cigarettes to the illness that had led to the patient's admission, although we did find that the gastroenterology unit had the highest prevalence of alcohol abuse, as might have been predicted. The small number of AUDIT positive cases would not have allowed any meaningful statistical investigation of this question.

## CONCLUSION

The results of this study are best viewed as a kind of needs assessment and feasibility study for the development and implementation of a liaison service designed to identify and treat patients with alcohol abuse and nicotine dependence.
